# Numerical Evaluation and Comparison of Kalantari's Zero Bounds for Complex Polynomials

**DOI:** 10.1371/journal.pone.0110540

**Published:** 2014-10-28

**Authors:** Matthias Dehmer, Yury Robertovich Tsoy

**Affiliations:** 1 Department of Computer Science, Universität der Bundeswehr München, Neubiberg-München, Germany; 2 UMIT - The Health & Life Sciences University, Department for Biomedical Informatics and Mechatronics, Hall in Tyrol, Austria; 3 Image Mining Group, Institut Pasteur Korea, Bundang-gu, Seongnam-si, Gyeonggi-do, Republic of Korea; Queen's University Belfast, United Kingdom

## Abstract

In this paper, we investigate the performance of zero bounds due to Kalantari and Dehmer by using special classes of polynomials. Our findings are evidenced by numerical as well as analytical results.

## Introduction

The problem of calculating the zeros of polynomials has been at the core of various algorithmic problems in engineering, computer science, mathematics, and mathematical chemistry [1]–[5]. One the one hand, determining all zeros of a complex polynomial explicitly has been crucial for practical problems [6–7]. One the other hand, estimations (bounds) for the moduli of real and complex zeros have been important for many reasons. For example, sharp zero bounds can serve as starting values for numerical procedures to calculate the zeros explicitly as already mentioned above. Also, zero bounds have been proven useful when estimating eigenvalues of matrices [8], [9].

We emphasize that numerous papers and books have been contributed dealing with the problem of locating the zeros of complex polynomials, see, e.g., [1]–[5], [10], [11]. Many papers thereof discuss the problem of determining disks in the complex plane where all zeros of a complex polynomial are situated. In view of the vast amount of existing zero bounds, their optimality has only been little investigated. In fact, many of the bounds which have been used extensively in practice do not give the precise annulus containing all zeros of a given polynomial. Also, sharpness results do not exist for all bounds which are practically to use.

In this paper, we deal with the problem of evaluating the quality of zero bounds numerically. A successor of this paper is [12]. In [12], we have put the emphasis on evaluating the quality of known bounds such as the ones due to Joyal, Mohammad, Kojima and Kalantari, see [12]–[16]. Another paper dealing with evaluating the quality of zero bounds numerically is due to McNamee and Olhovsky [17] who also evaluated classical and Kalantari's bounds on a set of polynomials with random real or complex roots. More precisely, they implemented 45 zero bounds for estimating the zeros with maximal modulus. These bounds have been evaluated on 1200 polynomials with random real or complex roots [17].

The main contribution of this paper is as follows: We focus on evaluating zero bounds developed by Kalantari [16] and Dehmer [1], [18] solely. In [17], it was claimed that some of the Kalantari's bounds are optimal on the mentioned set of polynomials. We show that some of the proposed bounds outperform Kalantari's bounds on special classes of polynomials. That proves it can be worthwhile to consider special classes of polynomials and special bounds which have been developed to operate on these classes. Examples for such bounds can be found in [18]. Also, we derive some analytical conditions to compare bounds due to Dehmer and Kalantari by means of inequalities, see, section ‘Numerical Results and Interpretation’.

## Methods

In the following, we state the zero bounds for locating the zeros of complex polynomials as theorems we will explore in this paper. The numerical results will be presented in the section ‘Results’.

### Kalantari and Dehmer Bounds

**Theorem 1 (Kalantari [16]).** *Let* 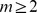 *and let* 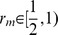 *be the positive root of the polynomial*

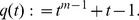
(1)


*For* 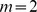 *and* 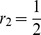, *all zeros of the complex polynomial*

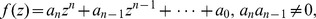



*lie in the closed disk*

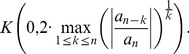
(2)


**Theorem 2 (Kalantari [16]).** *Let* 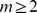 *and let* 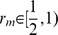 *be the positive root of the polynomial*

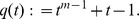



*For* 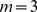 *and* 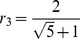, *all zeros of the complex polynomial*

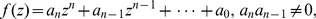



*lie in the closed disk*

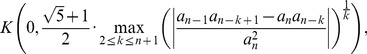
(3)

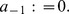



**Theorem 3 (Dehmer [18]).** *Let*

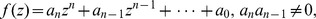



*be a complex polynomial. All zeros of* 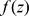 *lie in the closed disk*

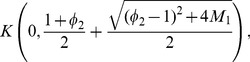
(4)


*where*

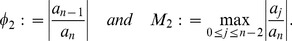
(5)


The next theorem gives a bound for polynomials with restrictions on the coefficients. Dehmer [1] has shown that such bounds can be more precise and often lead to better results when locating the zeros of polynomials. See also Table 3.

**Table 3:** Ratios for the polynomials by using Definition 3; 2≤*n*≤9.

	2	3	4	5	6	7	8	9
Kalantari, Th. (1)	1.739731	1.676344	1.633205	1.613733	1.618263	1.616748	1.623021	1.620896
Kalantari, Th. (2)	1.44096	1.392649	1.365094	1.348481	1.351714	1.354525	1.350528	1.351485
Dehmer, Th. (3)	1.627128	1.463874	1.424159	1.411194	1.418361	1.425269	1.441339	1.446148
Dehmer, Th. (4)	1.394486	1.495732	1.571549	1.630066	1.665533	1.696626	1.739386	1.777438

**Theorem 4 (Dehmer [18]).** *Let*

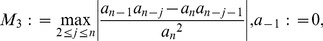
(6)


*and*

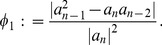
(7)


*In addition, let*

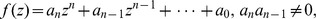



*be a complex polynomial. All zeros of* 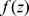 *lie in the closed disk* 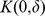 *where* 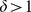 *is the largest positive root of the equation*

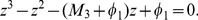
(8)


*Moreover,*

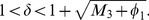
(9)


**Theorem 5 (Dehmer [18]).** *Let*

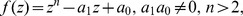



*be a complex polynomial. All zeros of* 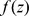 *lie in* 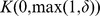 *, where* 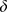 *is the unique positive root of the equation*

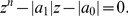
(10)


**Theorem 6 (Dehmer [18]).** *Let* 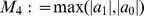 *and let*

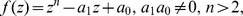



*be a polynomial with arbitrary coefficients. All zeros of* 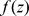 *lie in* 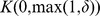 *, where* 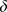 *is the unique positive root of the equation*

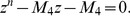
(11)


In [18], the following upper bound for these lacunary polynomials (see Theorem 6) has been stated without proof. Next, we here prove this result by assuming that the coefficients are positive and real-valued.

**Theorem 7.** *If the polynomial* 
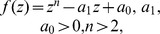
 *has two positive zeros, its largest positive zero* 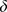 *satisfies*

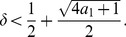
(12)


**Proof.** Since 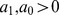 we infer by using the Descartes' rule of signs [10] that 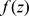 has either 2 or no positive zeros. We see that 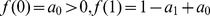 and 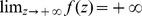. If 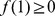, it follows that 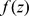 must have two positive zeros. The largest one is denoted as 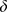 and we obtain 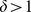. In order to get an estimation for 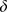, we consider
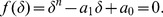
(13)


By using the finite geometric series, we obtain
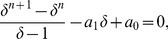
(14)and
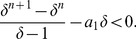
(15)This inequality leads to
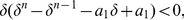
(16)and finally to



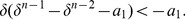
(17)However, this yields
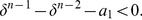
(18)


In order to get an inequality for 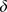, we set 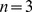. We get
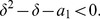
(19)


Determining the zeros of the latter function gives
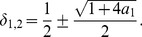
(20)


As
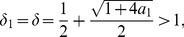
(21)we only consider the largest positive zero of the two. Now we define



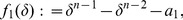
(22)

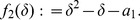
(23)


If we can prove that the positive zero of 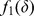 does not fall outside the interval 
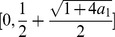
, we obtain Inequality 12. For this, we must prove that 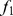 is strictly monotonically increasing in a certain interval.

Applying the Descartes' rule of signs to 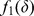 yields that its positive zero is unique. Also, 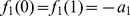 and 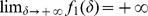. To prove the monotonicity, we consider
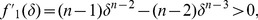
(24)that leads to



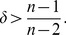
(25)As we here assume 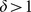, we see 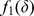 is strictly monotonically increasing for 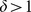. Finally we now prove that
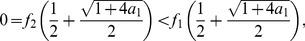
(26)hence,



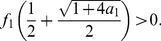
(27)Together with the monotonicity, that means that the positive zero of 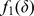 does not fall outside the 
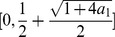
. We start with the inequality
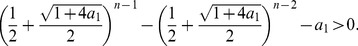
(28)


By performing elementary calculations, we get
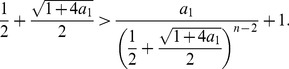
(29)


From this inequality, we also infer
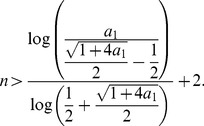
(30)


We finally show that the right hand side of this inequality is less than 4. That means claiming
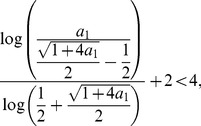
(31)


Yields
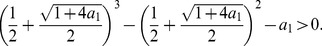
(32)


But by performing elementary calculations we find that this inequality is valid for 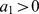. 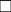


## Results

### Data: Classes of Complex Polynomials

As in [12], we define the classes of polynomials used in this study as follows. Note that the abbreviation ‘GD’ in the below stated definitions stands for *Gaussian Distribution*.

**Definition 1**

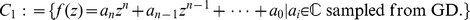
(33)


**Definition 2**

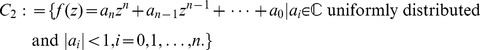
(34)


**Definition 3**

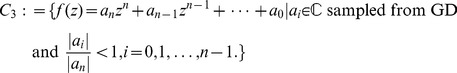
(35)


**Definition 4**

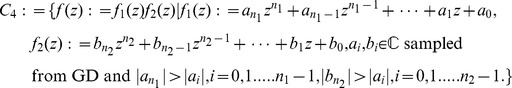
(36)


**Definition 5**

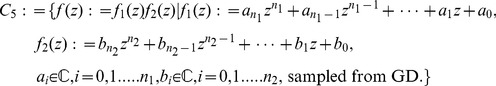
(37)


**Definition 6**

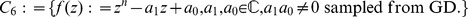
(38)


*These polynomials are called lacunary polynomials* *[4], [5]* *.*

### Statistical Analysis

In order to perform a statistical analysis, we have generated 1000 complex polynomials for each of the Definitions 1–6 and 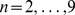. For each polynomial 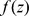, different bounds have been computed according to the Theorems 1–6. The following entity has been calculated:
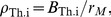
(39)where 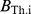 - bound value due to Theorem *i*, 
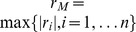
 - maximal modulus among the roots 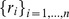 for the polynomial 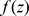. This entity reflects tightness of the bound, and its properties are:

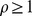
.
2. If 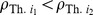, then the bound of Theorem *i*_1_ is tighter than the bound of Theorem *i*_2_.

**Figure 1:**
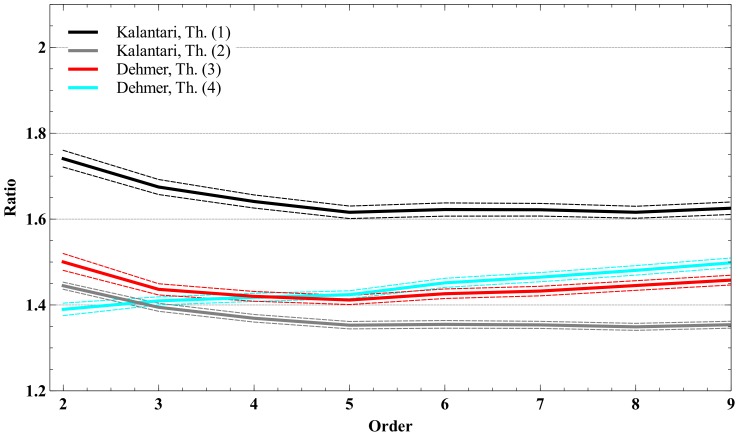
Bound ratios vs. polynomial order for Definition 1.

**Figure 2:**
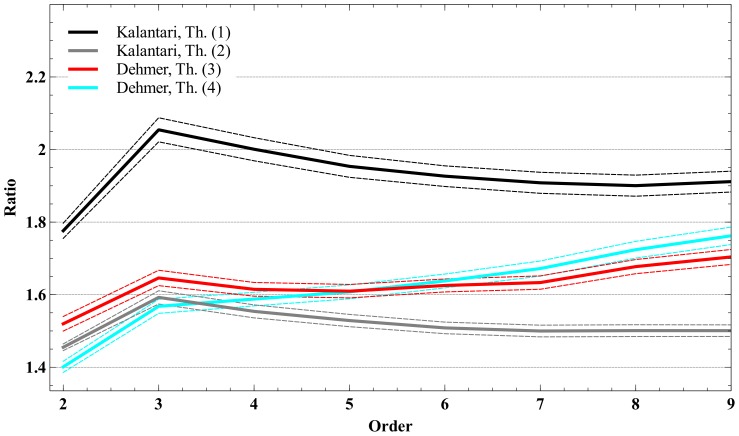
Bound ratios vs. polynomial order for Definition 5.

**Figure 3:**
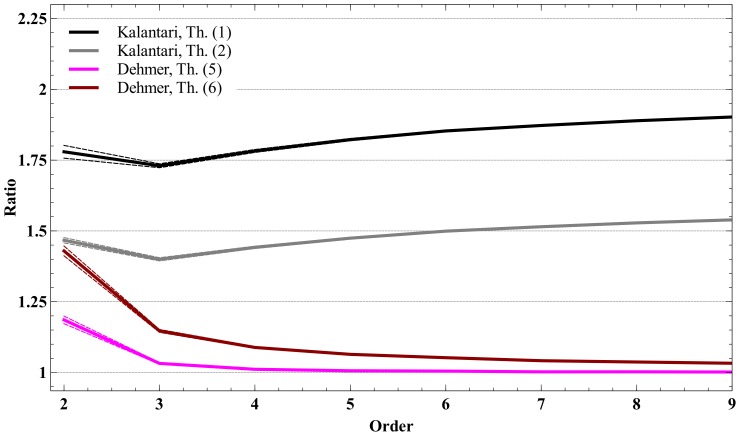
Bound ratios vs. polynomial order for Definition 6.

To compare different bounds averaged values of 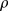 were calculated for a fixed 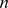 (Tables 1–6). The figures 1–3 illustrate the averaged bounds with 95% confidence intervals (dashed lines). The confidence intervals have been obtained by using two-sided *t*-test for 999 degrees of freedom:

**Table 1:** Ratios for the polynomials by using Definition 1; 2≤*n*≤9.

	2	3	4	5	6	7	8	9
Kalantari, Th. (1)	1.740618	1.674687	1.640986	1.615829	1.622275	1.62177	1.615899	1.625303
Kalantari, Th. (2)	1.444768	1.39455	1.36902	1.352937	1.354811	1.353599	1.349038	1.354005
Dehmer, Th. (3)	1.500152	1.436359	1.420059	1.411527	1.42618	1.432393	1.445271	1.45789
Dehmer, Th. (4)	1.449222	1.566359	1.634031	1.673176	1.732903	1.770081	1.807097	1.831334

**Table 2:** Ratios for the polynomials by using Definition 2; 2≤*n*≤9.

	2	3	4	5	6	7	8	9
Kalantari, Th. (1)	1.704931	1.625397	1.582924	1.572831	1.554937	1.556792	1.553397	1.566236
Kalantari, Th. (2)	1.429949	1.37537	1.357003	1.342865	1.340357	1.335035	1.330336	1.33636
Dehmer, Th. (3)	1.483377	1.432982	1.398882	1.39568	1.381683	1.390759	1.400006	1.413897
Dehmer, Th. (4)	1.369496	1.483964	1.517359	1.548124	1.551111	1.563088	1.596057	1.609

**Table 4:** Ratios for the polynomials by using Definition 4; 2≤*n*≤9.

	2	3	4	5	6	7	8	9
Kalantari, Th. (1)	1.750715	2.012142	1.837759	1.838256	1.823758	1.820873	1.810426	1.83248
Kalantari, Th. (2)	1.45379	1.572551	1.459837	1.460034	1.454432	1.461645	1.445665	1.457515
Dehmer, Th. (3)	1.609873	1.749532	1.566365	1.562589	1.56622	1.581601	1.58269	1.629539
Dehmer, Th. (4)	1.388793	1.773258	1.722926	1.783884	1.859031	1.979154	2.005109	2.168481

**Table 5:** Ratios for the polynomials by using Definition 5; 2≤*n*≤9.

	2	3	4	5	6	7	8	9
Kalantari, Th. (1)	1.776129	2.05439	2.000812	1.953628	1.926602	1.908316	1.900434	1.911558
Kalantari, Th. (2)	1.455061	1.592503	1.553934	1.528648	1.508396	1.499813	1.500933	1.500791
Dehmer, Th. (3)	1.519732	1.646073	1.614704	1.609604	1.625371	1.633324	1.677303	1.703949
Dehmer, Th. (4)	1.449809	1.841836	1.90772	2.015138	2.123091	2.222602	2.429363	2.520859

**Table 6:** Ratios for the polynomials by using Definition 6 (lacunary polynomials); 2≤*n*≤9.

	2	3	4	5	6	7	8	9
Kalantari, Th. (1)	1.779496	1.730165	1.782391	1.822496	1.853114	1.872392	1.889178	1.902321
Kalantari, Th. (2)	1.467053	1.399733	1.441984	1.47443	1.499201	1.514797	1.528377	1.53901
Dehmer, Th. (5)	1.185785	1.03211	1.011294	1.00614	1.004918	1.002322	1.002466	1.001667
Dehmer, Th. (6)	1.429314	1.146872	1.088454	1.064075	1.052297	1.041634	1.037083	1.032652



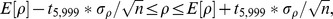
where 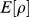 and 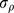 - are average and standard deviation for 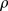; 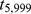 - *t*-distribution value for 95% two-sided critical regions with 999 degrees of freedom.

The pairwise comparison of the averaged values 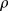 has been performed by using paired *t*-test. As a result we see that in the majority of cases, the values of 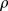 for the Theorems 1–6 are statistically different.

### Numerical Results and Interpretation

We restrict our analysis to evaluate the performance of the bounds due to Kalantari and Dehmer only, see, section ‘Methods’. In order to do so, we employ the classes of polynomials represented by Definitions 1–6.

#### General polynomials

We start by interpreting the Tables 1–5 and see that Kalantari's bound given by Theorem 1 is often worse than the zero bounds due to Dehmer, except the bound given by Theorem 4. Lets consider the polynomials of Definition 1 as this class is quite general. Except Theorem 4, the mean ratios of the bounds due to Dehmer are smaller than the ones by using Kalantari's bound given by Theorem 1. In particular this holds for Theorem 3 as well. Also, we observe that Theorem 2 due to Kalantari is optimal for *n*>4 when using the Definitions 1–3; by using the Definitions 4–5, we obtain the optimality for *n*>3. We emphasize that the results for Definition 6 (lacunary polynomials) will be discussed separately. In summary, this does not mean that no special polynomials exist whose evaluation may give the opposite result.

The analytical comparison of the bounds has been intricate. That means it might be difficult to compare bounds which rely on different concepts (e.g., explicit vs. implicit bounds, see [18]). Zero bounds are explicit if their values represent functions of the polynomial coefficients [18]. In contrast, a zero bound is called implicit if the value of the bound is a positive zero of a concomitant polynomial [18]. For instance, Theorem 1 and Theorem 3 are explicit but the Theorems 4–6 are implicit.

In case of using the explicit zero bounds Theorem 1 and Theorem 3, it is straightforward to derive an analytical expression (condition) to compare the bounds by means of inequalities. If we start with the inequality (i.e., we assume that Theorem 1 is better than Theorem 3),
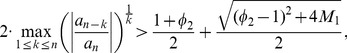
(40)we derive



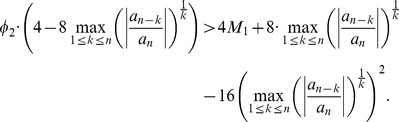
(41)If 
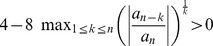
, then we finally get the condition
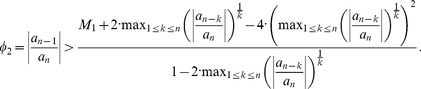
(42)


Otherwise, we yield
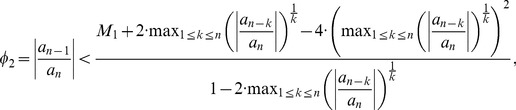
(43)with 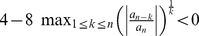. These inequalities can be used to compare Theorem 1 and Theorem 3 by means of inequalities assuming that Theorem 1 is worse than Theorem 3. Such a condition seems to be useful as we see by Tables 1–5 that the mean ratios of Theorem 3 are less than the ones by using Theorem 1.

To get an inequality for the assumption that Kalantari's bound given by Theorem 2 is better than Dehmer's bound given by Theorem 3, we start with assuming
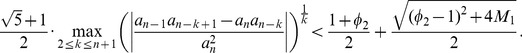
(44)


We yield
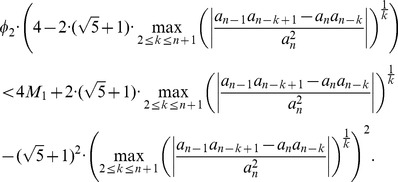
(45)


If 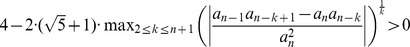, we obtain
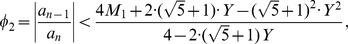
(46)with



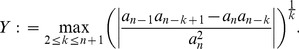
(47)Otherwise, we infer
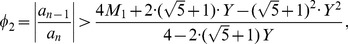
(48)with 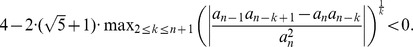 We note that all these inequalities can be evaluated explicitly and, hence, the corresponding conditions (inequalities) may be useful in practice.

#### Lacunary polynomials

The results of the evaluation for lacunary polynomials (see Definition 6) can be seen in Table 6. Dehmer's bounds given by Theorem 5 and Theorem 6 which have been designed for lacunary polynomials outperform both Kalantari bounds. For example, if we evaluate Theorem 1 and Theorem 2 for the polynomials of Definition 6), we obtain
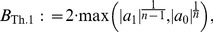
(49)and



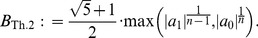
(50)Note that 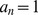, 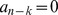, 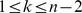. So, we see that these bounds differ by a constant factor only. The bound of Theorem 5 becomes to
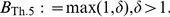
(51)


According to Theorem 7, an upper bound for 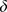 is 
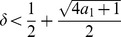
 if 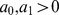. Note that this bound does not depend on 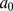. If 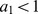, we infer 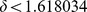. We observe that we always obtain 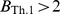 if 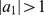 or 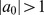. When considering Theorem 2, we always get 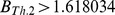 if 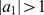 or 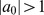. Even if 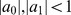, but the degree of the polynomials tends to be very large, the bounds of Theorem 1 and Theorem 2 tend to 2 and 1.618034, respectively. In summary, we see that the bound for lacunary polynomials due to Dehmer (see Theorem 6) gives often tighter bounds; in particular when *a*_1_<1. Similar arguments can be applied when considering Theorem 6.

## Summary and Conclusion

In this paper, we explored the performance of zero bounds due to Kalantari and Dehmer. In earlier contributions, it has been claimed [17] that Kalantari's bounds are often better than classical zero bounds. A similar study has been performed by Dehmer and Tsoy [12] who evaluated classical and more recent zero bounds for complex and real polynomials as well.

The main result of this paper is that some of the bounds due to Dehmer outperform the bounds due to Kalantari for special classes of polynomials. In particular when using lacunary polynomials (i.e., many coefficients equal zero) Dehmer's bounds showed excellent performance. We have underpinned our discussion to interpret the numerical results by analytical results. In particular, we have proved an upper bound for lacunary polynomials (see Theorem 7) and obtained conditions for some special cases to check whether one bound is better (or worse) than another by means of inequalities.

Another interesting line of research is to study the zeros of graph polynomials. Some recent related work dealing with applications on graph polynomials are [19]–[21]. In these contributions, graph polynomials have been used to encode special graphs, e.g., chemical graphs and also exhaustively generated networks. Consequently their zeros could be studied in terms of investigating structural properties of networks, see [22]. Zero bounds may play an important role to estimate the moduli of the underlying polynomials efficiently and to use these quantities for discriminating networks or to explore structural properties such as branching [20], [23], [24].
